# Comparison of Chinese inpatients with different types of medical insurance before and after the 2009 healthcare reform

**DOI:** 10.1186/1472-6963-14-443

**Published:** 2014-09-29

**Authors:** Shan Wang, Lihua Liu, Lin Li, Jianchao Liu

**Affiliations:** Department of Epidemiology and Biostatistics, School of Basic Medicine, Peking Union Medical College/Institute of Basic Medicine, Chinese Academy of Medical Sciences, Beijing, 100005 China; Institution of Hospital Management, Chinese PLA General Hospital, Beijing, 100853 China

**Keywords:** Basic medical insurance, Hospitalization cost, Average length of stay, China

## Abstract

**Background:**

Since 1994, China has established three major basic medical insurance (MI) schemes that aim to provide greater financial protection to members. The 2009 Chinese medical reform emphasized the enhancement of basic medical insurance. This study aims to investigate changes in hospital services costs for inpatients with different types of MI before and after the new Chinese medical reform.

**Methods:**

A total of 532,120 inpatient medical records, completed by 11 different hospitals nationwide in 2008 and 2011, were collected from the Ministry of Health retrospectively. Median and mean values were calculated to describe costs and average length of stay, respectively. A chi-square test was used to compare the distribution of patient visits. Wilcoxon rank-sum tests were conducted to compare costs.

**Results:**

The number of patients hospitalized increased. The average cost per stay in the three basic MI schemes increased, while out-of-pocket (OOP) spending decreased (P < 0.0001). The average cost per day showed similar trends. The purchase of Western medication accounted for the largest proportion of costs in all MI schemes in both years; however, these ratios decreased from 2008 to 2011, while those for other social insurance and OOP patients almost doubled. The average length of stay remained unchanged, and the average lengths of stay in the MI schemes differed before and after the healthcare reform.

**Conclusions:**

Healthcare reform with multipartite policies may make interactional impacts on hospitalization services for patients enrolled in MI schemes.

## Background

Affected by the economic system reforms in the 1970s, the Chinese medical care system was once criticized for a massive reduction in MI coverage, substantial increases in residents’ out-of-pocket (OOP) expenses, and a lack of fairness caused by the increasing gap between urban and rural areas and growing national health expenditure [1]. Facing these challenges, the government prepared new guidelines for healthcare reform in 2006 and officially launched them in 2009. The main aim was to ensure that basic healthcare services would benefit the public [2].

However, beginning in the 1990s, China gradually introduced a number of pilot projects and formally established basic social medical insurance to cover different sections of the population. The insurance was categorized into three main types of scheme: *Urban Employee’s Basic Medical Insurance* (UEBMI) for urban employees underwent a trial run in 1994, was officially issued in 1998, and aimed to provide MI for the urban working population; the *New Rural Cooperative Medical System* (NCMS), which was officially implemented in 2003, was a voluntary system intended to provide financial subsidies for rural residents’ MI; and *Urban Resident Basic Medical Insurance* (URBMI), which was established in 2007 and aimed to cover urban residents who were not employed [3]. In the development period following their establishment, these basic MI schemes continued to expand their coverage areas, and levels of protection were constantly improved.

The 2009 reform was comprehensive and involved multiple aspects of healthcare (e.g., financing, payment systems, pricing regimes, and organization of public hospitals). Nevertheless, definite measures were not constructed in a fixed manner but were established according to constant exploration of different provinces and cities. The first of five major targets in the 2009 reform plan focused on basic medical insurance, and the two main objectives were wider insurance coverage and higher funding levels [4]. Consideration of changes in costs and hospital service use by members of different MI schemes could reflect policy defects and the need for ongoing reform.

According to the Statistical Bulletin and Yearbook published by the Ministry of Human Resources and Social Security and Ministry of Health from 2008 to 2010, the national coverage of basic MI for urban workers, urban residents, and NCMS increased from 80.7% to 92.4%, 63.8% to 92.9%, and 90% to 96.6%, respectively [5]. Previously implemented free medical care and labor-protection medical care were gradually replaced by these social medical care insurance schemes. It should be noted that MI schemes designed under different systems provided different incentives, thus influencing the behavior of multiple interest groups such as patients, doctors, and hospitals [6]. With the expansion of insurance coverage and an increase in funding levels, other problems, aside from the provision of financial protection, could emerge. According to the aggregated data from the Health Statistical Yearbook, the costs per outpatient visit and inpatient stay continued to increase at a faster rate. For example, from 2005 to 2008, the cost per outpatient visit increased by 11.4 Yuan, while in the subsequent 3 years (2008–2011), it grew by 41.5 Yuan. The same circumstance can be observed in the cost of an inpatient stay, which showed a 572.6 Yuan increase in 2005–2008 but a 1,398.1 Yuan increase during 2008–2011 [7].

MI is generally expected to relieve financial pressure certain sections of the population and increase the use of health services [8–11]. However, it could generate the phenomenon of demand induction or patients’ excessive requests for medical services [12]. Moreover, as each population of MI scheme members presents different characteristics, specific reimbursement stipulation varies and may directly affect the extent to which patients seek medical services and the services provided by the healthcare system [13]. An example of this is the distinction between different schemes in terms of numbers of outpatients and inpatients [3]. Therefore, the effects of MI require long-term tracking and evaluation.

This study aimed to use first-hand clinical patient data from hospitals to observe changes in the extent of hospitalization of members of MI schemes before and after the implementation of the new healthcare reform. Inpatient visits, average inpatient cost per stay, average inpatient cost per day, and average duration of stay were measured in 2008 (before the health care reform) and 2011 (after the health care reform) and compared according to patient membership in each MI scheme, in order to obtain evidence regarding the evaluation of MI reform.

## Methods

### Study design and data sources

The study design was based on a retrospective comparative study. The data were taken from the cover pages of 532,120 inpatient medical records, which were completed by 11 hospitals in seven provinces in 2008 and 2011 and collected by the Ministry of Health. Of the seven provinces, three were middle and eastern provinces (Shandong, Hunan, and Jilin Provinces) and four were western provinces (Yunnan, Guangxi, Chongqing, and Sichuan Provinces). Of the 11 hospitals, 6 were tertiary and 5 were secondary. To protect patients’ privacy, their identities were concealed and only their medical record numbers used. These provinces and hospitals were not randomly selected; therefore, they are only very small examples of the overall numbers. However, as the sample size reached a certain extent, we hoped that analysis based on these regional hospitals would provide clues and perspectives to facilitate a more formal and large-scale reform assessment. This study was conducted in accordance with the declaration of Helsinki. This study was conducted with approval from the Ethics Committee of Chinese PLA General Hospital. Written informed consent was obtained from all participants.

### Measurement of variables

The variables measured were inpatient visiting times, inpatient costs per stay, structures of costs per stay, inpatient costs per day, and average length of stay. There were differences in these variables before and after the health care reform, and a comparison was conducted to compare the different MI schemes. The costs were obtained from patients’ bills, which were provided by the hospitals and recorded according to service category. Patients with different types of MI should pay their bills according to pre-set co-payment rules. The cost categories were bed use, nursing, Western medication, traditional medication, laboratory tests, diagnosis costs, surgery, and examination. The types of MI schemes included UEBMI, URBMI, NCMS, other social insurances, commercial medical insurance (CMI), and OOP.

### Statistical analysis

The variables of interest were described statistically and compared. The number of patient visits in the secondary and tertiary hospitals were grouped according to 2008 and 2011 MI scheme type. A chi-square test was used to compare differences in the distribution of members of various MI schemes between 2008 and 2011. The total inpatient cost was calculated, and average cost per stay and cost per day were described using the median due to the skewed distribution of the data. Moreover, Wilcoxon’s rank-sum test was performed several times to compare inpatient cost per stay and cost per day separately for each type of MI scheme between 2008 and 2011. The structure of costs per stay were analyzed to calculate the composition ratio for each charge subcategory for each MI scheme type. The mean value of the average length of stay was also calculated. Considering inflation, we adjusted all cost data in terms of the consumer price index (CPI) and altered monetary values for 2011 to correspond with those of 2008 (CPI_2011_/CPI_2008_ = 106.85). The CPI information was obtained from the National Bureau of Statistics of China website [7]. SAS software version 9.3 was used to conduct the statistical analyses. Because this was a descriptive study, cause–effect relationships were not established; rather, the analysis was intended to provide clues.

## Results

### Inpatient visits

Relative to those recorded in 2008, the total number of inpatient visits in the 11 hospitals in 2011 increased from 223,957 to 308,163, an increase rate of 37.6%. The highest rate of increase observed was 165.6%. The rates of increase in the secondary and tertiary hospitals were 57.1% and 36.0%, respectively (See Tables 1 and 2). However, discrepancies were observed according to MI scheme type. Numbers of UEBMI participants increased, with the number of hospitalizations roughly doubling. Among the patients hospitalized in 2008, there were only approximately 500 URBMI participants, whereas in 2011, this number reached more than 16,000. The number of NCMS patients decreased by 30% over the three years, and the number of OOP patients increased slightly. Regardless of hospital hierarchy, the structures related to hospitalized patients enrolled in different MI schemes showed statistically significant differences between 2008 and 2011 (Table 3).

**Table 1:** **Patient visits of secondary and tertiary hospitals, 2008 and 2011**

Year	Secondary	Tertiary	Total
2008	16746	207211	223957
2010	26312	281851	308163

**Table 2:** **Patient visits of each hospital, 2008 and 2011**

Year	H1	H2	H3	H4	H5	H6	H7	H8	H9	H10	H11	Total
2008	19608	3133	5283	3510	4811	18448	3142	60663	24449	25784	55126	223957
2011	36836	8321	8662	5491	5919	27463	6240	86600	37379	28455	56797	308163

**Table 3:** **Patient visits among different health insurance groups in secondary and tertiary hospitals, 2008 and 2011**

Health Insurance	Secondary hospitals				Tertiary hospitals			
	2008	2011	*χ* ^2^	*P*	2008	2011	*χ* ^2^	*P*
UEBMI	3686	2781	7124.99	<.0001	39609	78756	86394	<.0001
URBMI	125	363			364	16829		
NCMS	10645	9384			117659	87576		
Oth SI	1290	4259			13820	19969		
OOP	999	7674			35682	42141		
Total	16745	24461			207134	245271		

Note: Patients not classified to above groups were not included in the computation.

### Inpatient cost per stay and cost per day

Having adjusted costs according to the CPI, the total overall cost of the hospitalization of patients in all 11 hospitals increased from 2.1 billion Yuan in 2008 to 3.7 billion Yuan in 2011, an increase rate of 77.2%. The cost per stay increased from 4,985 to 6,478 Yuan, an increase rate of 29.9%.

Discrepancies were found in cost per stay for different MI scheme types between the two years. Cost per stay for members of the three basic MI schemes increased, whilst that of OOP patients declined dramatically. The rates of increase for UEBMI, URBMI, and NCMS were 28.7%, 33.1% and 18.2%, respectively. In contrast, this figure decreased by 43.4% for OOP patients. All of the differences were statistically significant (P < 0.0001). Cost per stay to patients enrolled in other social insurance schemes decreased slightly but were significantly different. However, there were no significant differences in costs per stay for individuals enrolled in commercial insurance (Table 4).

**Figure 1:**
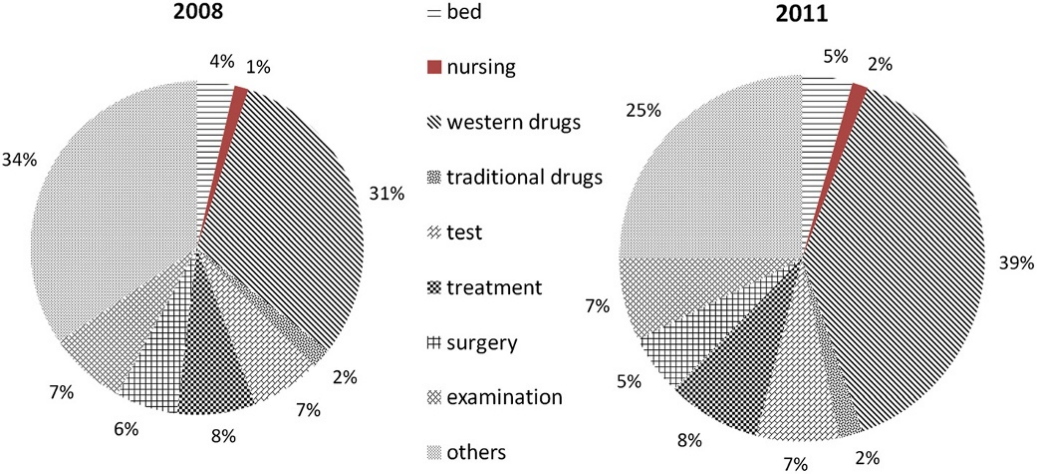
**Structure of inpatient cost per stay, 2008 and 2011.**

Examination of the overall picture of the 11 hospitals included in the study showed that the costs for all of the service categories increased from 2008 to 2011. In general, the category that increased at the fastest rate was Western medication (79.6%), increasing from 1,196 to 2,144 Yuan, whilst the category with the lowest rate of increase was surgery service (19.4%), increasing from 1,265 to 1,443 Yuan. However, it should be noted that while the rate at which nursing costs increased appeared to be substantial at 101.0%, the absolute increase was small (from 31 to 63 Yuan). The structure of cost per stay changed little between 2008 and 2011. In addition to a fast rate of increase, the Western medication category accounted for the largest proportion of cost per stay in both 2008 and 2011; this proportion increased from 30.6% in 2008 to 38.8% in 2011. Nursing, surgery, and traditional medication accounted for the lowest proportions of cost per stay at 1.6%, 6.2% and 2.4% in 2008 and 1.7%, 5.5% and 2.4% in 2011, respectively (Figure 1).

**Figure 2:**
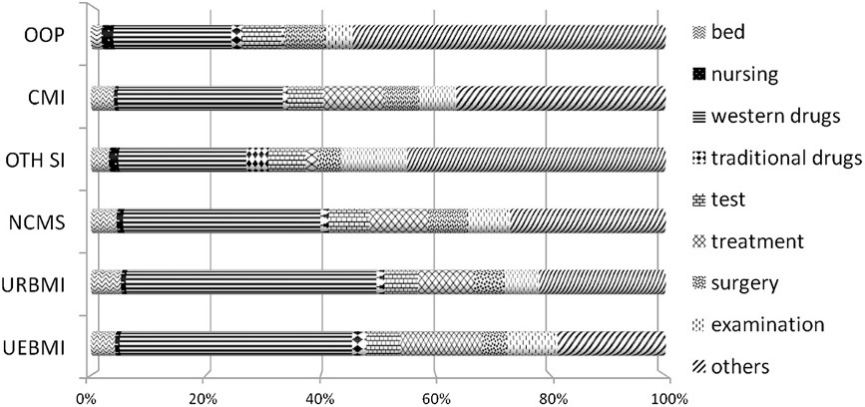
**Structure of inpatient cost per stay among different health insurance groups, 2008.**

Regardless of the type of MI scheme, the largest proportions of average hospitalization costs were accounted for by Western and traditional medication (Figure 2). For example, in 2008, the cost of Western medication in the UEBMI scheme accounted for 40% of cost per stay and that in URBMI was 46.5%. Western medication costs were 34% and 17% in CMI and OOP, respectively. Nursing and surgery showed small ratios regardless of MI scheme.

**Figure 3:**
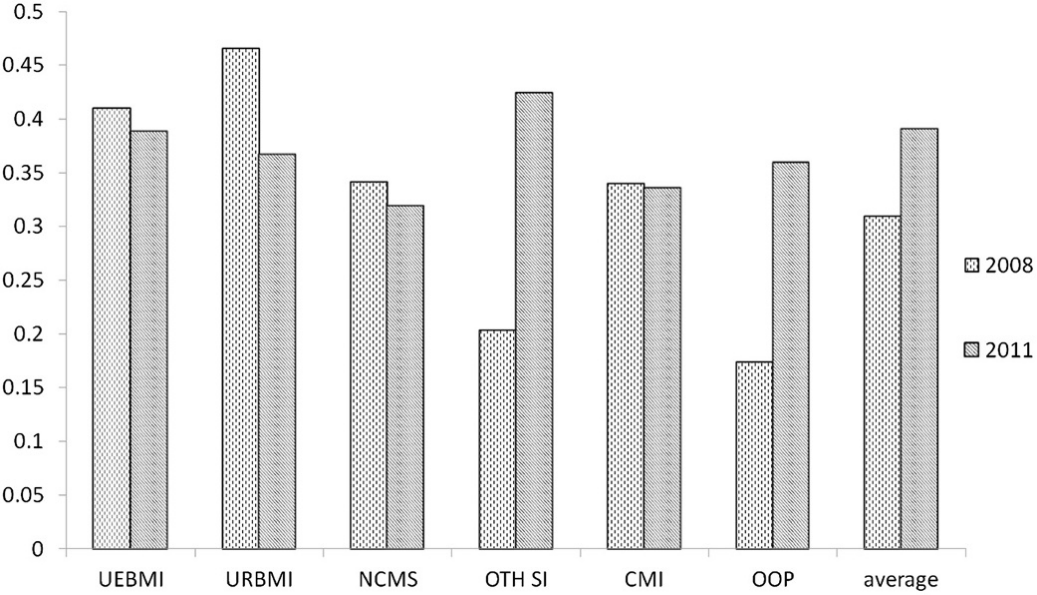
**Proportion of western drugs in hospital charges among different health insurance groups, 2008 and 2011.**

Further analysis was conducted with a focus on Western medication, which accounted for the largest proportion of cost. The proportions of western medication in UEBMI, URBMI, and NCMS patients declined and were lower than the average for all MI scheme members in 2011, whereas the opposite was true in 2008. In contrast, the proportion of Western medication costs to patients with other social insurance and OOP doubled. The Western medication cost to patients with commercial MI remained unchanged (Figure 3).

**Table 4:** **Inpatient cost per stay among different health insurance types, 2008 and 2011(CNY)**

Health insurance	2008		2011		P value
	n	charges	n	charges	
UEBMI	42660	5693	77419	7326	<0.0001
URBMI	476	3487	16822	4642	<0.0001
NCMS	127187	3656	86819	4320	<0.0001
OTH SI	15007	10286	19898	10031	<0.0001
CMI	77	5051	1381	5104	0.9989
OOP	36506	8378	41842	4740	<0.0001

Cost per day increased from 574 Yuan in 2008 to 752 Yuan in 2011, an increase rate of 31.0%, which was a similar finding to that for cost per stay. The reason for this result may be that the average length of remained stable. The comparison between MI categories revealed that, aside from CMI, the cost per day to members of other MI schemes differed significantly (Table 5).

**Table 5:** **Day cost among different types of health insurance, 2008 and 2011 (CNY)**

Health insurance	2008		2011		*P* value
	N	Day cost	N	Day cost	
UEBMI	42660	548	77419	708	<0.0001
URBMI	476	377	16822	606	<0.0001
NCMS	127187	492	86819	598	<0.0001
OTH SI	15007	883	19898	957	<0.0001
CMI	77	588	1381	567	0.4834
OOP	36506	909	41842	642	<0.0001

### Average length of stay

The average length of stay for all inpatient visits plateaued before and after the reform. A difference in duration of stay was observed between secondary and tertiary hospitals. The average length of stay increased from 9.0 to 10.0 days in the secondary hospitals following the reform, and that in the tertiary hospitals remained at 12.5 days.

Before and after the reform, the average length of stay for MI scheme members in the secondary hospitals in 2008 was 15 days or fewer. However, the average length of stay for URBMI patients had increased by 4.9 days and reached 18.6 days in 2011, and that of UEBMI patients increased to 15 days. The average length of stay for OOP patients increased from 6.8 days to 10.1 days from 2008 to 2011. In 2008, no information regarding commercial insurance in the secondary hospitals was recorded; however, in 2011, the average length of stay for patients with commercial insurance in the secondary hospitals was exceptionally high, reaching 28.1 days. In the tertiary hospitals, the average length of stay for UEBMI and NCMS patients remained unchanged and decreased by 4.2, 3.9, 2.5 and 0.4 days for URBMI, other social insurance, CMI, and OOP patients, respectively.

## Discussion

**Figure 4:**
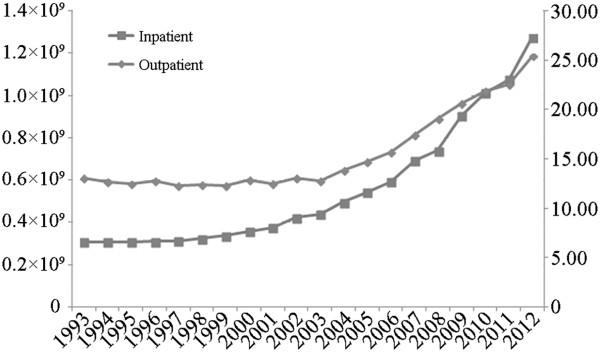
**Outpatient visits and numbers of admissions, 1993–2012.** Source: Chinese Health Statistics Yearbook 2012.

The total number of inpatient hospital admissions increased significantly between 2008 and 2011. Despite the obvious distinctions in rates of increase between various MI schemes, the study’s findings were generally consistent with the trend reported in national health statistics over the same period, as shown in Figure 4. These statistics presented an accelerating upward trend in both outpatient visits and inpatient hospital admissions subsequent to the establishment of the first new basic MI scheme in China in 1994. It reasonably suggests that this trend is closely related to the fact that the newly established basic MI scheme continued to expand its coverage and gradually provided considerable financial protection and fulfilled the healthcare needs of residents, which resulted in a reduction in their unmet needs in a broader sense. The diverse rates of increase in for patients enrolled in the different MI schemes may be associated with the difference in the populations covered by the individual MI schemes and the ways in which they operate. For instance, UEBMI, which covered urban workers, serves a population with relatively high incomes. In contrast, the URBMI was designed for the unemployed urban population, which predominantly consists of women, older adults, and children. Other empirical evidence is likely to provide insight into the reasons for the results. For example, Chinese public hospitals have developed rapidly over the past few decades, and the scale of hospital entities has continued to expand. The number of hospitals with more than 800 wards rose from 488 in 2008 to 976 in 2011 [7]. One recent study suggested that the provision of hospital beds may function according to Roemer’s law (i.e., any beds provided will always be filled) [14]. However, this study was unable to determine whether the results observed in the sample hospitals adhered to Roemer’s law.

The rate of increase in the total cost of inpatient admissions in the 11 hospitals included in the study (91.6%) mirrored the national trend during the same period (96.1%; without CPI adjustment) [7]. The cost per stay for members of the three newly established MI schemes increased by 18% to 33%, and those of CMI and OOP patients neither increased nor declined. Supplier-induced consumption and moral hazards to consumers are considered possible underlying reasons that cannot be ruled out in any insurance issue [15]. That is, the impact of different MI schemes may differ with respect to the behavior of those involved in both demand and supply. Members of the three basic social insurance schemes tended to seek excessive medical services, which resulted from poor awareness of costs under the financial protection of basic MI schemes. Hospitals may provide unnecessary medical services to avoid increasing the financial burden to patients. Moreover, fee-for-service payment methods in the healthcare system provided strong incentives for such behavior.

A similar inclination was apparent in the costs of Western medication. Western medication costs accounted for a large proportion of costs in each basic MI scheme but a relatively small proportion in CMI and OOP cases. Hospitals may prescribe the “big prescription” to patients with basic social MI to increase their revenue or meet patients’ requirements [16]. However, this study was not able to determine the effects based on the available data. Despite the facts outlined above, the proportion of cost per stay accounted for by Western medication costs for members of the three basic MI schemes decreased from 2008 to 2011, but it increased for OOP patients. According to the Statistical Yearbook of the Ministry of Health, the proportion of national cost per stay accounted for by medication costs decreased from 43.5% to 41.8% during the same period [7], which is consistent with the findings of the current study. The yearbook suggested that MI schemes may bear speculation risks but can be employed appropriately as selective reimbursement policy tools to control medication use. In fact, the combined implementation of the 2009 Essential Drugs List functioned well and achieved certain benefits [17]. However, numerous unresolved difficulties with medication circulation require further regulation. Basic MI schemes alone are not likely to be a powerful tool in controlling rising medication costs [18–20].

In sharp contrast to the high proportion of Western medication costs, those of nursing and surgery were relatively small regardless of the MI scheme type. In addition, the situation remained stable before and after the reform. However, under fee-for-service payment, nursing and surgery directly reflected the labor value of medical staff in all categories of charges. Pricing of surgery and nursing care, as critical parts of hospital services consuming professional knowledge and manpower skill resources, were low and did not correctly reflect the value of the care. In contrast, Western medication and examinations with low labor costs but substantial industrial product, material or device consumption constituted a great expense. The distortion in the pricing system with low labor costing provided negative incentives for medical professionals in hospitals to obtain compensatory benefits through high-cost procedures [18]. In this regard, risks may be increased. For instance, excessive examinations and the “big prescription” were constantly present. Consequently, the quality of medical care could be compromised and the motivation for professional improvements weakened. Evidently, to achieve reasonable and steerable healthcare expenditure, the pricing system requires rearrangement. Prices for pharmaceuticals, materials, and devices should be down regulated, and the prices of consultation, surgery, and nursing care should be increased appropriately to reflect the labor, technical, and risk-bearing value of medical staff.

The average length of stay (ALOS) is usually calculated by summing hospitalization days for each patient and dividing the total by the number of inpatients. Though the number of hospitalizations increased substantially in 2011 relative to those observed in 2008, the ALOS basically remained stable. The suggested underlying reason was that hospitals had increased their bed capacity, as discussed above. In terms of the difference between secondary and tertiary hospitals, the ALOS of for all MI schemes in the secondary hospitals increased, but it remained unchanged or decreased in tertiary hospitals. This finding could be connected to the remarkable disparities in rates of bed use between the tertiary and secondary hospitals. According to the National Statistical Yearbook, the rate of bed use in tertiary hospitals in 2011 was 104.2%, whilst those of secondary and primary hospitals were 88.7% and 58.9%, respectively [11]. The policy defects underlying this could be those of a lack of function differentiation between tertiary and secondary hospitals and the absence of an effective referral system [21]. The first consultation, treatment, and the rehabilitation process were completed in one medical institution, in most cases a high-level hospital, even if the cost was relatively high. Therefore, tertiary hospitals were overcrowded, whereas the small hospitals were deserted. In light of the growing waiting list, high-level hospitals should consider ways to reduce the ALOS, thus expediting ward turnover. In contrast, small hospitals should seek ideas to increase the ALOS, as this may be a means of increasing revenue, particularly with financial protection provided by basic social health insurance schemes.

## Conclusions

MI scheme type is one of the factors affecting inpatient costs in various patient groups. Moral hazards to suppliers and consumers are more likely to be observed in three basic MI schemes, namely URBMI, URBMI, and NCMS. Hospital level is also an influential factor. Moreover, the interactions between health policies, such as those for deficient payment methods, distorted price regulation, lack of a referral, system, and an immature medication distribution system, also play a role. In summary, MI reform is one of the most significant achievements in China’s new round of healthcare reform. It has facilitated the provision of medical care. To ensure the effectiveness of MI schemes in the long term, a comprehensive policy that includes simultaneous cooperative reforms of various health policies is urgently required in ongoing healthcare reform; however, this would lead to a complicated system and require long-term assessment.
